# Diseases of the Kidney

**Published:** 1906-01-20

**Authors:** 


### PROGRESS IN MEDICINE AND SURGERY.

### DISEASES OF THE KIDNEY.

**Nervous System and Bright's Disease.**—In a useful article on this subject Leszynsky [1] has summarised and grouped the chief symptoms referable to the nervous system which occur in Bright's disease, especially chronic interstitial nephritis. Whatever view is taken of the pathology of this complicated condition, two facts may be held responsible for the nervous disorders which occur—firstly, the circulation of toxins; and secondly, malnutrition of the nervous centres owing to arteriosclerosis. Leszynsky believes that, whereas in some cases renal degeneration, by impeding the elimination of poisonous substances, sets up vascular degeneration, in others the arterial changes are the cause of the chronic granular kidney: in any given case it may not be possible to say to which class the condition is referable; nor is it always possible to assign a definitely toxæmic or a definitely nutritional cause for the nervous symptoms; but as a rule the more serious symptoms, such as permanent hemiplegia or sudden death from hæmorrhage are due to arteriosclerotic changes. The transient objective phenomena, on the other hand, are usually toxic in origin. Headache, throbbing, or a sense of pressure or fulness in the head occur, which are similar to those described by neurasthenics. Though generally occipital and bilateral, no special type of headache is characteristic; somnolence, nausea, vomiting, vertigo, and mental confusion sometimes occur, which with optic neuritis may be mistaken for symptoms of meningitis or tumour. Migraine may have pre-existed, or may be simulated in some cases of chronic nephritis. The vomiting may closely simulate that occurring in intracranial disease. Supraorbital neuralgia, cutaneous hyperæsthesia, and various rheumatic pains may also be attributed to a wrong cause. Attacks of blindness are of two kinds—those in which the pupillary light reflex and the fundus oculi remain normal, and those when the pupils fail to react to light, and there is stasis of the optic papillæ. The former class often recover suddenly and spontaneously. Uræmic blindness is commoner after attacks following scarlatina and pregnancy. Deafness is the only other special sensory disturbance which is at all common. Muscular twitchings are common, and definite convulsions resembling major epilepsy or Jacksonian epilepsy may occur. The temperature in these cases may be either subnormal or raised, and the diagnosis must rest on the history and condition of the urine and cardiovascular system. Coma may be the only and first symptom of uræmia, and these cases are often diagnosed as cerebral hæmorrhage. Ephemeral attacks of paralysis may be caused by thrombosis, vascular spasm, or cerebral œdema; but in all the cases which have been described degeneration of the cerebral arteries has been found post-mortem; that this is the main factor in the causation of so-called uræmic paralysis is shown also by the fact that similar attacks occur in persons suffering from endarteritis, from other causes apart from renal disease. Mental symptoms occasionally occur, such as delirium, hallucinations, disorientation, etc., but they present nothing characteristic, and renal disease is not an important element in the causation of insanity. The paper serves well to call attention to the importance of examining elderly patients with obscure nervous symptoms with a view to detecting chronic interstitial nephritis. With this object, besides frequently testing the urine for albumen, the amount, specific gravity, and the total urea and solids should be determined, and compared with the amount of food taken and the general condition of the metabolism.

**Albuminuria without Nephritis.**—That nephritis may exist, at any rate in the pathological sense, without albumen being at any time detected in the urine is well known; out of a series of 581 cases of nephritis collected by Stengel, no albumen was found at any time in 215.[2] Medical opinion is also tending towards the converse view, namely, that albuminuria may occur apart from nephritis. Two very instructive and important papers have recently appeared which bear on this question. One is a contribution from the clinical point of view by Dukes.[3] The other, by A. E. Wright and Ross,[4] deals rather with the pathological and laboratory side of the matter. Dukes has, during the last 35 years, investigated the albuminuria of adolescents, the so-called cyclic, functional, postural or physiological albuminuria, as exemplified in a large number of boys at a great public school. He estimates that between the ages of 13 and 14, 16 per cent. of boys suffer from albuminuria—the largest proportion occurring in September, after the summer holidays. This albuminuria presents certain characteristic features. It tends to increase during the period of school life; it is remarkably capricious in its occurrence in the same patient, being present in large amount one day and perhaps absent on the next; it can be usually completely controlled by either rest in bed on ordinary diet, or by milk diet while the patient is up and about; it may, apparently, continue for many years. Dukes, in 1891, thought that " a considerable proportion of these cases never recover, but progressively increase "; but further experience and observation of numbers of men who as boys suffered from albuminuria have compelled him to change his opinion. The cases, he considers, may be divided into three classes. The largest number present a pulse of high tension, this increased tension, however, being very variable from day to day, like the albuminuria. Excess of nitrogenous food, imperfect action of the " scavengers of the body," and a hereditary tendency to gout are the principal factors in its production, and it may be controlled by alkalies, an occasional blue pill, restriction of nitrogenous food, and attention to mastication and proper rest after meals. The next class are those with cold, clammy, swollen extremities, a large feeble soft pulse, cardiac dilatation, and liability to chilblains even in summer. The treatment for these is plenty of food, especially proteids, and tonics such as strychnine and arsenic. The third class consist of thin neurotic subjects, and the treatment is to secure a smooth, even way of life without undue strain or excitement. Bromides, with occasional blue pill, are valuable, exercise without competition should be insisted on, but work and play may be allowed to continue as long as harrassing examinations and competitions are avoided. From his large clinical experience Dukes concludes that in all these classes the albuminuria is due to faulty adjustment of the cardiovascular system to the blood-pressure, so that the nerves fail to control the blood-vessels effectively, and allow the renal capillaries to become congested and transude serum albumen into the urinary tubules. He regards it, therefore, as a vascular neurosis dependent on the unstable conditions of youth and growth, to be met by proper regulation of the mode of life as regards work, exercise, food, etc. He has no doubt that with reasonable treatment these cases recover on attaining maturity, and are then no more liable to actual Bright's disease than other people. From a rather different point of view Wright and Ross practically arrive at the same conclusion. Setting aside the question of albumen, they determine the functional efficiency of the kidney by estimating its power to excrete salts and the ratio which the percentage of salts in the urine bears to the percentage of salts in the blood. This ratio they call the " excretory quotient." The method of determining these percentages of salts is interesting and original. The disintegration of red blood cells in a solution of salts depends on the amount of salts present, and thus by using progressive dilutions of urine or blood it is possible to find the minimum dilution of each which will effect complete hæmolysis. By comparing the dilutions so obtained the salt content of the respective fluids can also be compared; or in other words, the excretory quotient can be determined. In eight typical cases of " physiological albuminuria " it was found that the excretory quotient was always high, and fully equal to that found in normal persons, so that it was presumed that the kidneys in physiological albuminuria are functionally unimpaired. The next point determined, by experiment on six cases, was that by giving calcium chloride, which increases the coagulability of the blood, the albuminuria in " functional " cases could be markedly restrained, whereas in four cases of renal disease no such effect was observed. In this connection it is to be noted that milk diet increases the lime content in the body fluids and the coagulability of the blood. After enumerating the clinical features of cases of albuminuria of the " physiological " variety and comparing them with those of urticaria, Wright and Ross conclude that both effects are due to vascular instability, leading to increased tension in the capillaries and transudation of serum, either into the renal tubules or the deep layers of the skin. By increasing the coagulability of the blood, by rest and other means, this condition may be controlled, and a diagnosis may in the renal cases be readily made by observing the effect of calcium chloride. They are in agreement with Dukes in considering physiological albuminuria no bar to an active and prolonged life, and no reason for refusing or loading up cases presenting themselves for life assurance.

[1] Med. Rec., May 20, 1905, p. 765. [2] Ibid., July 29, 1905, p. 196. [3] Brit. Med. Jour., Oct. 7, 1905, p. 848. [4] Lancet, Oct. 20, 1905, p. 1164.

(*To be continued.*)

